# Effect of 2-Methylthiazole Group on Photoinduced Birefringence of Thiazole-Azo Dye Host–Guest Systems at Different Wavelengths of Irradiation

**DOI:** 10.3390/molecules27196655

**Published:** 2022-10-07

**Authors:** Beata Derkowska-Zielinska, Anna Kozanecka-Szmigiel, Dariusz Chomicki, Vitaliy Smokal, Yutaka Kawabe, Oksana Krupka

**Affiliations:** 1 Institute of Physics, Faculty of Physics, Astronomy and Informatics, Nicolaus Copernicus University in Torun, Grudziadzka 5, 87-100 Torun, Poland; 2 Faculty of Physics, Warsaw University of Technology, 75 Koszykowa Str., 00-662 Warszawa, Poland; 3 Faculty of Chemistry, Taras Shevchenko National University of Kyiv, 64/13 Volodymyrska St., 01601 Kyiv, Ukraine; 4 Chitose Institute of Science and Technology, 758-65 Bibi, Chitose 066-8655, Hokkaido, Japan; 5 Micro et Nanomédecines translationnelles (MINT), University of Angers, French National Institute of Health and Medical Research (INSERM) 1066, CNRS 6021, F-49000 Angers, France

**Keywords:** thiazole–azo dye, photoinduced birefringence, thin films

## Abstract

The photoinduced birefringence behaviors of host–guest systems based on heterocyclic thiazole–azo dyes with different substituents, dispersed into PMMA matrix, were investigated under three excitation wavelengths, i.e., 405 nm, 445 nm or 532 nm. The wavelengths fell on the blue side, near the maximum or on the red side of the absorption bands of *trans*-azo dyes, respectively. We found that photoinduced birefringence was generated at a similar extent in all studied systems, except the system containing a 2-methyl-5-benzothiazolyl as thiazole–azo dye substituent. For this material, the achieved birefringence value was the highest among the whole series, regardless of the excitation wavelength. Moreover, we identified the optimal irradiation wavelength for efficient birefringence generation and showed that large absorption of excitation light by *trans* isomer does not account for achieving a significant degree of molecular alignment. The obtained results indicate that thiazole–azo dye with a 2-methyl-5-benzothiazolyl substituent shows promising photoinduced birefringence, and can be considered a dye potentially suitable for optical applications.

## 1. Introduction

In the last decades, light-responsive molecules have been of interest in many applications, such as optical data storage, optical switches, optical memory, etc., [1,2,3,4].

Azo dyes are the best known family of photoresponsive compounds, which have different properties and can also have a refractive index depending on the polarization and propagation direction of light, namely birefringence.

Azobenzenes attract the attention of the scientific community due to their photochromic nature, ease of processing and simplicity of design [5,6,7]. These features allow changing their physico-chemical properties for a particular application by appropriate modification of their chemical structure [8,9,10,11]. It is well known that the core of azo dyes is formed by the conjugated azo (-N = N-) chromophore group in combination with one or more aromatic or heterocyclic systems. The addition of electron withdrawing and/or electron donating substituents to the backbone of azo moiety can significantly influence the absorption spectra of azo dyes by affecting the reorganization of electronic density [12,13,14]. The D–π–A system of azo compounds can provide a prerequisite ground state charge asymmetry [15] as well as efficient intramolecular charge transfer (ICT) between donor and acceptor groups [16,17] because the π-conjugated bridge ensures a pathway for electronic charges movement [15]. The transition from the ground state to the excited state upon excitation causes almost instantaneous electronic polarization, which changes the dipole moment of the molecules and generates a dipolar push–pull system [16,17,18,19]. Therefore, substitution of one or more benzene rings with easily delocalizable electron-excessive and/or electron-deficient hetero-aromatic rings, acting as an auxiliary electron donors and/or acceptors, can result in enhanced intramolecular charge transfer [15,16,17].

The most important feature of azo compounds is a possibility of *trans–cis* photoisomerization, which can be induced and reversed depending on the wavelength of the incident light. It is well known that this photochromic behavior can differ due to the structure of studied compounds (i.e., location and the shape of π–π* and n–π* bands) [6,8,9,20,21].

Another interesting feature of azo compounds is the photoinduced orientation. Generation of optical anisotropy in azo dye-containing materials (e.g., azo dyes dispersed in polymer matrix or azopolymers) results from the orientation of azo chromophores induced by linearly polarized light due to processes of selective absorption and reactions of *trans–cis* isomerization [22,23]. After numerous *trans–cis–trans* isomerization processes, the long axes of azo molecules tend to align in directions perpendicular to the polarization of light. As a result, the material becomes birefringent and dichroic in the plane perpendicular to the direction of light propagation [6,24,25].

The efficiency and dynamics of the light-induced birefringence generation strongly depend on various factors, which are related to the chemical structure of azo dye (such as substituents of the azo group and its bulkiness), a chromophore content and a type of polymer matrix, but also with the experimental conditions (e.g., an excitation wavelength and intensity) [26,27,28]. While some general principles govern the efficiency of the light-induced processes in azo compounds, the optical response of a given material may be substantially different than expected [29,30].

A polymeric material with unique properties, such as light weight, high flexibility and low-cost of production, can be used to improve the quality of a prepared thin layer [31]. One of the most popular and widely used polymeric materials is poly(methyl methacrylate) (PMMA). Its main advantages are excellent mechanical properties, high chemical resistance, simple synthesis, low cost, good tensile strength, low optical loss in visible spectral range, good insulation properties and thermal stability. PMMA-based matrices are well known not only for their good optical transparency, but also for high resistance to laser damage [32,33]. PMMA is an excellent and suitable host material in the host-guest systems due to its optical clarity and known chemical and physical properties. It should also be added that for the samples based on PMMA it is possible to conduct research of the structure of matrices and photophysical transitions connected with changes in the mobility of low molecular structural units.

Studying the correlation between structure and material properties is a fascinating field of research, which is very important for the development of novel materials for specific applications such as optical data storage. Typically, measurements of photoinduced birefringence generation were carried out at a single excitation wavelength located on the red side of the azo moiety absorption band. However, the measurements performed for various excitation wavelengths may provide valuable information on the optimal experimental conditions leading to the most efficient process of azo chromophore alignment.

The aim of this work was to characterize the photoinduced birefringence generation in thiazole–azo dyes host–guest systems under irradiation with linearly polarized violet, blue or green light. The motivation for this research is the possibility of using thiazole–azo dyes in photonic devices for recording optical information (optical data storage), which are becoming increasingly important in many fields. In this article, we focus on the effect of an additional 2-methylthiazole group on the efficiency photoinduced birefringence generated in thiazole–azo dyes dispersed into a poly(methyl methacrylate) (PMMA) matrix. Furthermore, we introduce the benzene ring into a thiazole fragment in the position of 4, which can improve solubility of the compounds. The key structural feature of the investigated materials is the presence of a heterocyclic thiazole fragment in the azo molecule, which leads to the change in the distribution of the electron density of the conjugation system in comparison with azobenzenes without a heterocyclic fragment. We show that upon irradiation with polarized violet, blue or green light, the studied azo dye systems can exhibit photoinduced birefringence. To the best of our knowledge, the photoinduced birefringence generation for these heterocyclic thiazole–azo dyes dispersed into a PMMA matrix at 405 nm, 445 nm and 532 nm are presented for the first time.

## 2. Results and Discussion

### 2.1. UV-Vis Spectra

**Figure 1:**
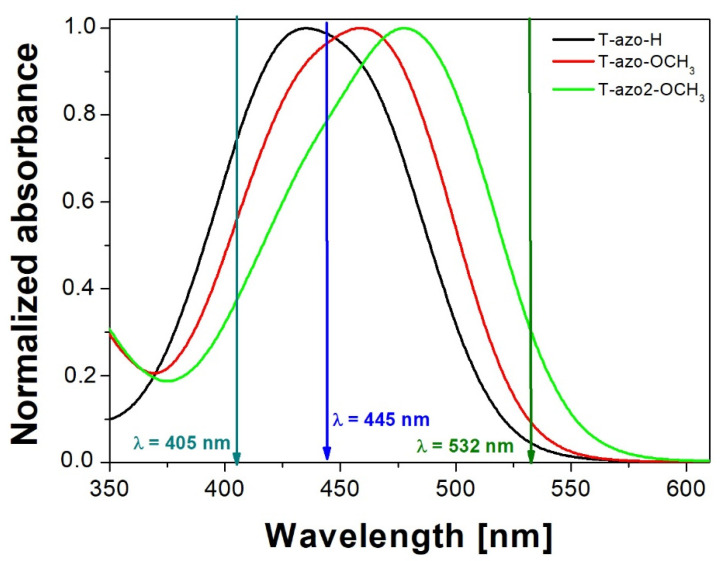
Normalized UV-Vis spectra of thiazole–azo dyes (molar concentration of dyes 27.2 mM) dispersed in PMMA matrix thin films: **T–azo2–OCH_3_**, **T–azo–OCH_3_**, **T–azo–H** with thickness 877 nm, 1128 nm, 1312 nm, respectively. The arrows indicate the absorbance of studied samples at the excitation wavelengths used.

Figure 1 shows the UV-Vis spectra of the studied thiazole–azo dyes dispersed in the PMMA matrix thin films (**T–azo–OCH_3_**, **T–azo2–OCH_3_**, **T–azo–H**). One can see that the π–π* and n–π* bands are completely overlapped in this region, and the absorption bands of **T–azo–OCH_3_**, **T–azo2–OCH_3_** samples are redshifted relative to the thin film of **T–azo–H** without any substitution in para-position [8]. We also found that the absorption band of **T–azo2–OCH_3_** thin film with 2-methyl-5-benzothiazolyl moiety is redshifted compared to **T–azo–OCH_3_** film with a phenyl ring.

It should also be noted that the excitation wavelength of 445 nm used in the photoinduced birefringence measurement was the most strongly absorbed by the examined samples compared to the excitation wavelengths of 405 nm and 532 nm. The samples were transparent at the probing wavelengths (690 nm or 783 nm, respectively).

**Figure 2:**
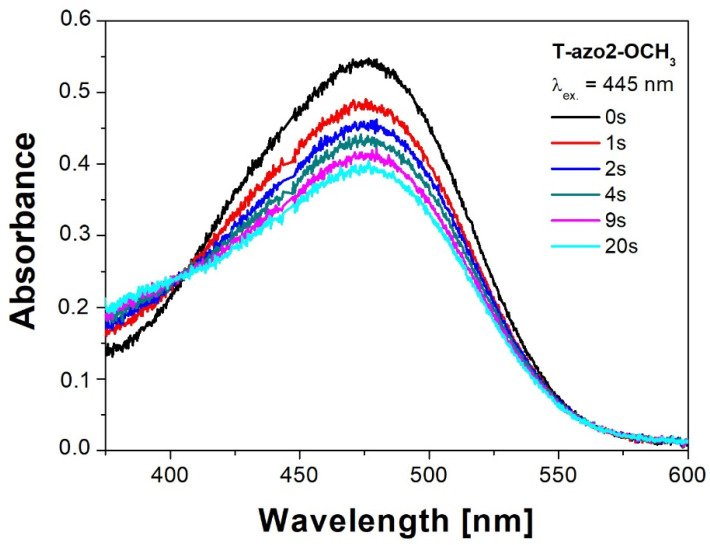
Absorption spectra of **T–azo2–OCH_3_** before (0 s) and during irradiation (1 s–20 s) with 445 nm.

Figure 2 presents the changes in the absorption spectra observed for the **T–azo2–OCH_3_** sample under irradiation with 445 nm light. The *trans–cis* isomerization process was confirmed by the presence of isosbestic points (at 405 nm for **T–azo2–OCH_3_**) and decrease in the *trans*-isomer band intensity.

### 2.2. Photoinduced Birefringence

**Figure 3:**
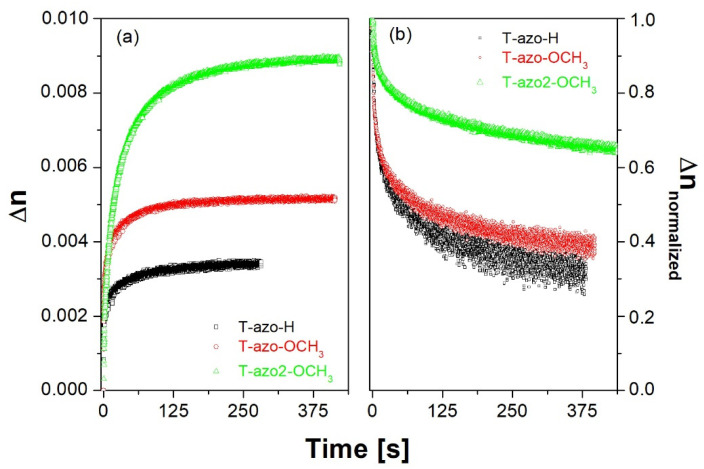
Birefringence (**a**) growth and (**b**) normalized relaxation curves for the thiazole–azo dyes dispersed in PMMA matrix thin films **T–azo–OCH_3_**, **T–azo2–OCH_3_**, **T–azo–H** (λ_exc._ = 405 nm, λ_probe_ = 690 nm).

Figure 3 present the birefringence growth and relaxation curves for the thiazole–azo dyes dispersed in PMMA matrix thin films, where *λ_exc_*_._ = 405 nm and *λ_probe_* = 690 nm were used. We found that the irradiation time of a few hundred seconds was already sufficient to observe saturation of birefringence in the studied compounds (see Figure 3a). We also found that thiazole–azo-PMMA samples **T–azo–OCH_3_** and **T–azo2–OCH_3_** have a higher saturation level of birefringence compare to the **T–azo–H** one without substituent in para-position. Moreover, **T–azo2–OCH_3_** with a heterocyclic fragment (R1) has the highest final birefringence, which is almost twice as that for **T–azo–OCH_3_** with a phenyl fragment (R1). At the same time, **T–azo2–OCH_3_** exhibits the most stable birefringence after irradiation among the series (Figure 3b). One can also see that the **T–azo–OCH_3_** sample with a phenyl ring (R_1_) and electron donating group (R_2_) has almost a similar birefringence value after relaxation with **T–azo–H** and it is almost two times smaller than for **T–azo2–OCH_3_**.

**Figure 4:**
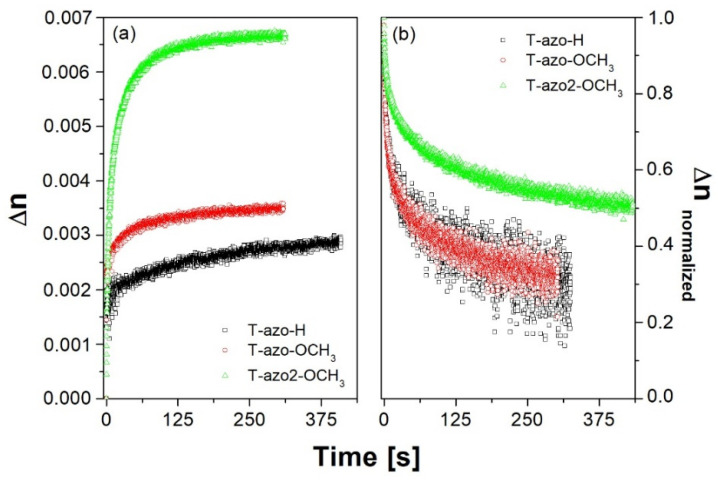
Birefringence (**a**) growth and (**b**) normalized relaxation curves for the thiazole–azo dyes dispersed in PMMA matrix thin films (λ_exc._ = 445 nm, λ_probe_ = 690 nm).

The curves of birefringence growth under 445 nm excitation and birefringence relaxation for the studied films are shown in Figure 4. It is interesting that despite a strong film absorbance at this wavelength, the values of final birefringence observed for all the samples were lower than the values obtained in the case of 405 nm excitation. The result may be explained on the basis of the recorded changes in the absorption spectra under irradiation. Both *trans-* and *cis*-isomers are involved in the process of optical birefringence generation, and thus, light absorption by the *cis* form is essential for obtaining a significant degree of molecular order. *Cis*-isomers more effectively absorb the 405 nm wavelength than 445 nm light, which compensates for the effect of a lower absorption of 405 nm light by *trans*-isomers.

As in the case of 405 nm excitation, we found that the highest birefringence, under 445 nm, was also induced in **T–azo2–OCH_3_** film (thiazole–azo dye with 2-methyl-5-benzothiazolyl substituent—R_1_ and methoxy group R_2_), which again correlates with the slowest birefringence relaxation rate (see Figure 4b). Its final birefringence is almost twice as large as the final induced birefringence compared to **T–azo–OCH_3_** due to an additional 2-methylthiazole group. We also found that host-guest film of thiazole–azo compound **T–azo–OCH_3_** with a phenyl ring (R_1_) and electron donating group (R_2_) demonstrate higher birefringence saturation level compared to **T–azo–H** without a substituent in para- position (R_2_). Figure 4b shows the normalized birefringence relaxation curves after turning off the beam at 445 nm. One can see that the type of substituent strongly affects the relaxation of birefringence. We found that **T–azo2–OCH_3_** exhibits the lowest relaxation, which may be associated with different geometry of chromophores **T–azo2–OCH_3_** vs. **T–azo–OCH_3_** and **T–azo–H** with more compact structure. It is difficult to relax the molecules to the isotropic state by thermal movement if the chromophores have a big volume. The 2-methyl-5-benzothiazolyl group in **T–azo2–OCH_3_** increases the steric effect and slows down the relaxation of birefringence of **T–azo2–OCH_3_**. However, a thiazole–azo compound with a phenyl ring (R_1_) and electron donating group (R_2_) (**T–azo–OCH_3_**) has similar birefringence to **T–azo–H**. Therefore, the role of various substituents in thiazole–azo dyes in the photoinduced birefringence measurements is evident.

**Figure 5:**
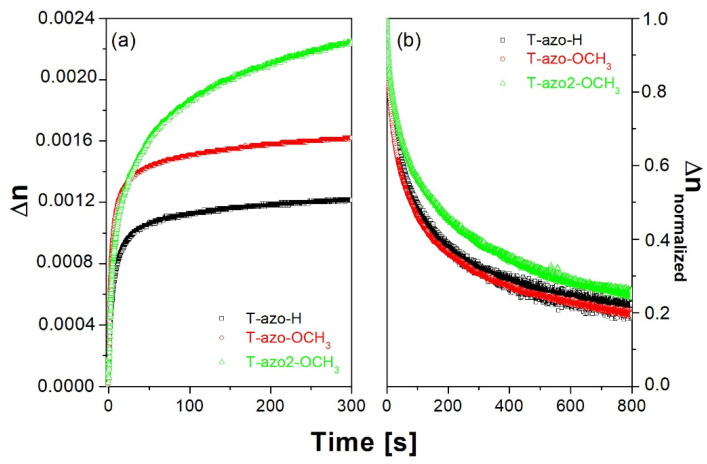
Birefringence (**a**) growth and (**b**) normalized relaxation curves for the thiazole–azo dyes dispersed in PMMA matrix thin films (λ_exc._ = 532 nm, λ_probe_ = 783 nm).

Figure 5 presents the birefringence growth and relaxation curves for the thiazole–azo dyes dispersed in PMMA matrix thin films, where *λ_exc_*_._ = 532 nm and *λ_probe_* = 783 nm were used. Apart from **T-–azo2–OCH_3_**, the irradiation time of about 300 s was sufficient to observe birefringence saturation for all samples. We also found that the highest birefringence saturation level was induced in **T–azo2–OCH_3_** with the highest absorption value at 532 nm. This can be explained by a significantly red-shifted absorption band and arising strongest absorption of 532 nm light among the series. Nevertheless, the values of photoinduced birefringence generated under 532 nm irradiation were very low. The result can be attributed to low sample absorbance, i.e., the excitation wavelength falls on the tails of *trans* absorption bands for all the samples.

From Figure 5a, it can be seen that the birefringence saturation level decreases as follows: **T–azo2–OCH_3_** > **T–azo–OCH_3_** > **T–azo–H**. Thus, the thiazole–azo compound with 2-methyl-5-benzothiazolyl substituent (R_1_) (**T–azo2–OCH_3_**) has a higher saturation level of birefringence compared to thiazole–azo dyes with a phenyl ring (R_1_). Similar behavior was visible in absorbance. For 532 nm excitation (see Figure 5b), the relaxation of birefringence was similar for all studied compounds.

**Figure 6:**
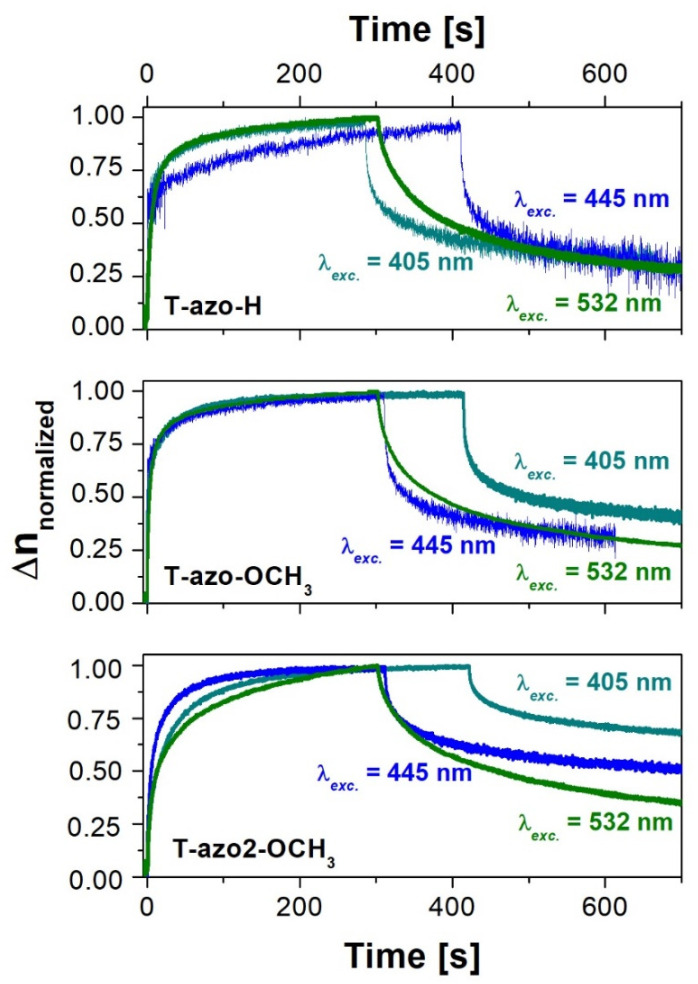
Birefringence growth and relaxation curves for selected thiazole–azo dyes dispersed in PMMA matrix thin films for 405 nm, 445 nm and 532 nm.

Figure 6 presents the examples of birefringence growth and relaxation curves for the thiazole–azo dyes dispersed in PMMA matrix thin films for three excitation wavelengths, i.e., 405 nm, 445 nm and 532 nm. In all cases, we observed the rapid increase of birefringence at the beginning of pumping (see Figure 6), which was due to the molecular arrangement orientation of the thiazole–azo dye, which gradually tended to be perpendicular to the polarization direction of the pumping light; thus the detecting light intensity began to increase gradually. Then we can see a slow increase to the saturation level with different speeds depending on the type of substituent. When the pumping light was turned off, the curves decreased sharply due to the molecular relaxation. The anisotropic state re-establishes the originally mixed and disordered distribution. However, this type of recovery is not complete, because some azo molecules achieve equilibrium, and some still remain at an orientation distribution state.

The decay of birefringence after turning off the excitation light was caused by the thermal *cis–trans* isomerization of thiazole–azo chromophores and a thermal randomization of the molecular orientation.

We found that the final birefringence generated after irradiation with 405 nm light was the highest for all studied thiazole–azo dyes. The difference between the increase in the birefringence for the studied wavelengths strongly depends on the type of substituents in thiazole–azo compounds.

The birefringence growth and birefringence relaxation with time are often described by the following biexponential equations [25]:

(1) $$\Delta n=A\left[1-exp\left(\frac{-t}{\tau_{1}}\right)\right]+B\left[1-exp\left(\frac{-t}{\tau_{2}}\right)\right]$$

(2) $$\Delta n=Cexp\left(\frac{-t}{\tau_{3}}\right)+Dexp\left(\frac{-t}{\tau_{4}}\right)+E$$

where *τ*_1_, *τ*_2_ are time constants for writing processes, *τ*_3_, *τ*_4_ are time constants for relaxation processes, *A*, *B*, *C* and *D* are amplitudes associated with different physical processes appearing upon illumination, and *E* is the residual birefringence.

Using Equations (1) and (2), one can perform the curve fitting, which allows us to quantitatively compare the obtained birefringence signals. It should be noted that the biexponential growth and biexponential relaxation reproduced the results of the experiment well. The values of the fitted parameter for **T–azo–H, T–azo–OCH_3_** and **T–azo2–OCH_3_** are presented in Table 1 and Table 2. The contributions of various processes to birefringence growth and relaxation were calculated using Equation (3):

(3) $$X_{in}=\frac{X_{i}}{\sum_{i}X_{i}}$$

where *X_i_* = *A*, *B* and *C*, *D*, *E* for birefringence growth and relaxation, respectively.

When excited with 405 nm light, for samples **T–azo–H** and **T–azo–OCH_3,_** the fast and slow processes contributions to the birefringence growth are the same for both materials and are 0.65 and 0.35, respectively. The time factors for both components are of the same order. For the sample **T–azo2–OCH_3_**, the slow component has more impact on birefringence growth than for the other materials, and the fast and slow processes’ contributions are 0.51 and 0.49, respectively. The time factors are noticeably longer than for the other two samples, especially for the slow component, whose time factor is one order of magnitude higher.

Upon excitation with 445 nm light, the fast process contribution to birefringence growth is higher than the slow process contribution. For the samples **T–azo–H** and **T–azo2–OCH_3_**, the fast and slow processes contributions are around 0.6 and 0.4, while for the sample **T–azo–OCH_3_** it is 0.74 and 0.26, respectively. The time factors for the fast process are the same order for **T–azo–H** and **T–azo–OCH_3_**, and for **T–azo2–OCH_3_** they are one order of magnitude higher. The time factor for the slow component is the lowest for the sample **T–azo2–OCH_3_**, and for **T–azo–H**, it is one order of magnitude higher than for the other samples.

When excited with 532 nm light, the fast and slow processes’ contributions to the birefringence growth are similar for **T–azo–H** and **T–azo–OCH_3_** samples, and their values are around 0.74 and 0.26, respectively. For the sample **T–azo2–OCH_3,_** the fast and slow processes’ contributions are both equal, and their value is 0.50. The time factors for the fast and slow processes are the same order of magnitude. The values of the time factors of the slow process are similar for the **T–azo–H** and **T–azo2–OCH_3_**, and it is the lowest for the **T–azo–OCH_3_** sample, while for the fast process, the time factors are similar for the **T–azo–H** and **T–azo–OCH_3_** samples, and it is slightly higher for the **T–azo2–OCH_3_** sample.

Table 2 shows the fitted parameters for the birefringence relaxation. After excitation with 405 nm light, the fast process contribution to birefringence relaxation is slightly higher than the slow process contribution. The sample **T–azo2–OCH_3_** exhibits the highest residual birefringence, while for the sample **T–azo–H**, it is the lowest. Both time factors for the fast and slow processes are the lowest for the **T–azo–H** sample, and they are the highest for the **T–azo2–OCH_3_** sample.

The fast processes contribution to birefringence relaxation after 445 nm excitation is slightly higher than the slow process contribution. Again, the sample **T–azo2–OCH_3_** exhibits the highest residual birefringence, and the sample **T–azo–H** has the lowest. Time factors are the same order of magnitude, and both time factors are the lowest for the **T–azo–OCH_3_** sample. They are the highest for the **T–azo2–OCH_3_** sample.

The fast process contribution to the birefringence relaxation is considerably higher than the slow process contribution after excitation with 532 nm light, for all the samples. The residual birefringence is similar, and it is around 0.1 of the maximum birefringence value. Time factors, separately, are the same order of magnitude. Both time factors are the lowest for the **T–azo–OCH_3_** sample. The fast process time factor is the highest for the **T–azo2–OCH_3_** sample, while the slow process time factor is the highest for the **T–azo–H** sample.

Table 3 summarizes the ratios between the maximum birefringence and absorbance for the given excitation wavelengths. As can be seen, there is no clear influence of the amount of the absorbed light on the maximum birefringence value. Even though the absorbance at 405 nm and 445 nm is the lowest for the **T–azo2–OCH_3_** sample, the birefringence values are the highest. For the 532 nm light, the absorbance of the **T–zo2–OCH_3_** sample is the highest amongst the three studied samples, and the birefringence value is the highest as well. However, the ratio between the two parameters is the lowest.

## 3. Materials and Methods

### 3.1. Chemical Structure

**Figure 7:**
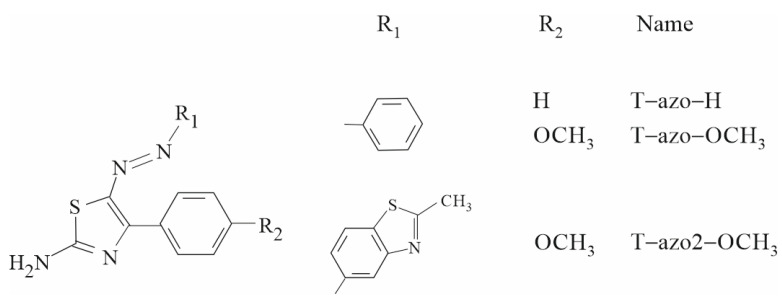
The molecular structures of studied thiazole–azo dyes.

Figure 7 shows the chemical structure of the studied thiazole–azo dyes. The synthesis procedure for **T–azo–H**, **T–azo–OCH_3_** and **T–azo2–OCH_3_** is described elsewhere [8,9,20,34]. ^1^H NMR (400 MHz) spectra were recorded on a Mercury (Varian) 400 spectrometer.

4-(4-Methoxyphenyl)-5-[(2-methyl-1,3-benzothiazol-5-yl)diazenyl]-1,3-thiazol-2-amine: dark red crystals, yield 80%. ^1^H NMR (400 MHz, DMSO-d_6_): *δ* = 2.83 (s, 3H, CH_3_), 3.88 (s, 3H, OCH_3_), 7.01 (d, *J* = 8 Hz, 2H, Ar), 7.75 (d, *J* = 7.2 Hz, 1H, Het), 7.88 (d, *J* = 7.2 Hz, 1H, Het), 8.13 (s, 1H, Het), 8.25 (d, *J* = 8 Hz, 2H, Ar), 8.49 (br. s, 2H, NH_2_) ppm.

4-Phenyl-5-(phenyldiazenyl)-1,3-thiazol-2-amine: Dark red crystals, yield 84%. ^1^H NMR (400 MHz, DMSO-d_6_): *δ* = 7.30 (t, *J* = 7.6 Hz, 1H), 7.40–7.47 (m, 5H), 7.63 (d, *J* = 7.6 Hz, 2H), 8.14 (d, *J* = 7.2 Hz, 2H), 8.42 (br. s, 2H, NH_2_) ppm.

4-(4-Methoxyphenyl)-5-(phenyldiazenyl)-1,3-thiazol-2-amine: Red solid residue, yield: 88%. ^1^H NMR (400 MHz, DMSO-d_6_): *δ* = 3.87 (s, 3H, OCH_3_), 6.99 (d, *J* = 7.2 Hz, 2H, Ar), 7.28 (t, *J* = 6.8 Hz, 1H, Ph), 7.41 (t, *J* = 6.8 Hz, 2H, Ph), 7.62 (d, *J* = 6.8 Hz, 2H, Ph), 8.23 (d, *J* = 7.2 Hz, 2H, Ar), 8.46 (br. s, 2H, NH_2_) ppm.

### 3.2. Preparation of Thin Films

The standard procedure was used to prepare thin films of studied thiazole–azo dyes dispersed in the PMMA (poly(methylmethacrylate)) matrix using a spin-coating method [8,9]. THF solutions including PMMA and the thiazole–azo dyes were prepared first. PMMA was purchased from Sigma-Aldrich and was used as it was. Films were formed on glass substrates using a spin-coating method with the spinning time of 60 s. After that, films were baked at 60 °C for 3 h in a vacuum chamber. The thickness of the samples was in the range of 900–1300 nm.

### 3.3. UV-Vis Absorption

The absorption spectra of all studied thin layers of heterocyclic thiazole–azo compounds dispersed in the PMMA matrix were measured with a spectrometer (Shimadzu UV-1800) in the range 350–600 nm.

### 3.4. Photoinduced Birefringence Measurements

**Figure 8:**
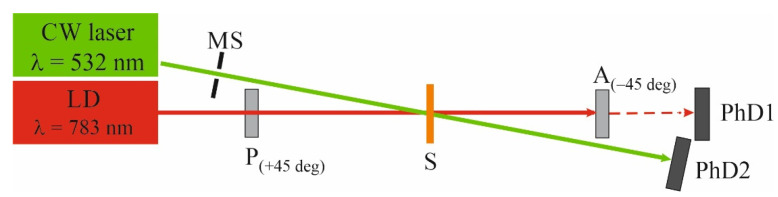
Photoinduced birefringence experimental setup at 532 nm: P—polarizer; A—analyzer; PhD—photodetectors; M—mirror; S—sample; MS—mechanical shutters.

Photoinduced birefringence measurements were performed for 405 nm, 445 nm and 532 nm excitation wavelengths. The experimental configuration used in the studies with violet and blue irradiation was presented elsewhere [35]. The intensity of each beam (from diode lasers) was 100 mW/cm^2^. The time-evolution of birefringence generation and birefringence decrease after switching on and off the excitation light was probed by 690 nm wave. The excitation and probe beams were linearly polarized in the directions forming an angle of 45°. The measurement technique is based on detecting the intensity of the probe beam after passing through the thin film situated between two crossed polarizers [35]. The details of the experimental configuration were described elsewhere [36,37,38], whereas Figure 8 shows the experimental configuration of photoinduced birefringence at excitation of a CW laser (λ_exc._ = 532 nm, 0.365 mW, I~29 mW/cm^2^). The details of this setup were described elsewhere [24].

## 4. Conclusions

The optical birefringence was induced in three heterocyclic thiazole–azo dyes with different substituents dispersed in a PMMA matrix, upon polarized violet, blue and green irradiation. We found that the role of the substituents in thiazole–azo dyes and irradiation wavelength is visible during the birefringence generation.

We noticed that the photoinduced birefringence response at 405 nm, 445 nm or 532 nm of most studied host-guest thin films of PMMA–thiazole–azo dyes with different substituents is similar, except for thiazole–azo dye with 2-methyl-5-benzothiazolyl substituent (i.e., **T–azo2–OCH_3_**). It was found that this molecule had the highest saturation level of birefringence compared to other studied thiazole–azo dyes for all three induced irradiation wavelengths (i.e., 405 nm, 445 nm and 532 nm). This dye also exhibited the lowest relaxation after ceasing the irradiation 405 nm and 445 nm wavelengths. We suppose that the high Δn value obtained for **T–azo2–OCH_3_**, despite its lower absorption at these wavelengths, can be attributed to the free space in the polymer, created by the bulky **T–azo2–OCH_3_** chromophores, giving them the opportunity to reorient.

The chemical structure is the main factor influencing the photoinduced behavior of the studied thiazole–azo dyes. The introduction of the thiazole–azobenzene unit into PMMA matrix, restricts the chromophore motions during the writing process. In the host–guest polymers, the chromophores are typically more mobile, which can induce a faster inscription of Δ*n*. Therefore, the appropriate design of thiazole–azo dyes can increase the properties of photoinduced birefringence, which contributes to their use in new photonic devices such as optical data storage.

## Figures and Tables

**Table 1:** Fitted parameters for the birefringence growths of **T–azo–H, T–azo–OCH_3_** and **T–azo2–OCH_3_** for 405 nm, 445 nm and 532 nm.

	T–azo–H	T–azo–OCH_3_	T–azo2–OCH_3_
		405 nm	
*A_n_*	0.65	0.65	0.51
*τ_1_* [s]	0.41	0.72	6.38
*B_n_*	0.35	0.35	0.49
*τ_2_* [s]	45.90	39.98	64.07
	445 nm		
*A_n_*	0.62	0.74	0.59
*τ_1_* [s]	0.08	0.13	3.22
*B_n_*	0.38	0.26	0.41
*τ_2_* [s]	137.70	54.80	40.31
	532 nm		
*A_n_*	0.73	0.75	0.50
*τ_1_* [s]	5.43	3.57	5.86
*B_n_*	0.27	0.25	0.50
*τ_2_* [s]	80.02	75.35	101.40

**Table 2:** Fitted parameters for the birefringence relaxation of **T–azo–H, T–azo–OCH_3_** and **T–azo2–OCH_3_** for 405 nm, 445 nm and 532 nm.

	T–azo–H	T–azo–OCH_3_	T–azo2–OCH_3_
		405 nm	
*C_n_*	0.40	0.30	0.14
*τ_3_* [s]	5.79	7.15	17.80
*D_n_*	0.29	0.26	0.21
*τ_4_* [s]	110.20	121.70	271.80
*E_n_*	0.31	0.43	0.65
	445 nm		
*C_n_*	0.41	0.34	0.19
*τ_3_* [s]	6.40	4.80	11.40
*D_n_*	0.31	0.31	0.27
*τ_4_* [s]	142.50	90.80	164.80
*E_n_*	0.28	0.34	0.53
	532 nm		
*C_n_*	0.60	0.54	0.56
*τ_3_* [s]	97.20	72.93	132.64
*D_n_*	0.30	0.38	0.33
*τ_4_* [s]	1200.00	813.90	1162.80
*E_n_*	0.10	0.08	0.11

**Table 3:** Maximum birefringence values, absorbance at the excitation wavelengths and their ratio for **T–azo–H, T–azo–OCH_3_** and **T–azo2–OCH_3_** samples.

	T–azo–H	T–azo–OCH_3_	T–azo2–OCH_3_
		405 nm	
Δn_max_	0.0034	0.0051	0.0089
Absorbance	0.7329	0.8815	0.4198
Δn_max_/Abs.	0.0046	0.0058	0.0212
	445 nm		
Δn_max_	0.0029	0.0035	0.0067
Absorbance	0.9743	1.5295	0.8983
Δn_max_/Abs.	0.0030	0.0023	0.0075
	532 nm		
Δn_max_	0.0012	0.0016	0.0022
Absorbance	0.0461	0.1526	0.3501
Δn_max_/Abs.	0.0260	0.0105	0.0063

## Data Availability

Data supporting the results of this study are available from the appropriate author upon reasonable request.

## Sample Availability

Samples of the compounds T–azo–H, T–azo2–OCH_3_ and T–azo–OCH_3_ are available from the appropriate authors upon reasonable request.
